# Creep evolution characteristics and deformation mechanisms of carbonaceous slate under the action of groundwater

**DOI:** 10.1038/s41598-025-91429-7

**Published:** 2025-03-07

**Authors:** Hucheng Yang, Peng Li, Shengrui Su, Jianxun Chen

**Affiliations:** 1 College of Geology Engineering and Geomatics, Chang′an University, Xi’an, 710054 China; 2 Department of Architecture and Engineering, Yan’an University, Yan’an, 716000 China; 3 Key Laboratory of Western China’s Mineral Resources and Geological Engineering of Ministry of Education, Chang′an University, Xi’an, 710054 China

**Keywords:** Carbonaceous slate, Creep strain, Pore characteristics, Morphology of layered surface, Civil engineering, Mechanical engineering, Rheology

## Abstract

The carbonaceous slate exhibits significant creep characteristics and large deformations during tunnel excavation, especially in groundwater-rich areas. Studying the creep characteristics and deformation mechanism of carbonaceous slate under the influence of groundwater is substantial and essential. Triaxial creep tests of dry and saturated rock samples at different bedding angles were performed to examine the deformation characteristics using the GCTS-RTX1000 test system. The layered surface morphology was analyzed before and after the creep to reveal the deformation mechanisms. The results showed that the softening effect of water causes a significant reduction in the strength of the carbonaceous slates. The transient deformation of saturated samples was greater than that of dry samples due to the water absorption in the pores at a low-stress state. Creep in the saturated rock samples is more pronounced in the first stage compared to the dry samples, and the gap decreases as the stress increases. In the creep processes, the saturated samples manifested the pressure dissolution, debris migration, and deposition in the stratified surface of the particle skeleton under the water and the stress. These results are helpful for better understanding the creep characteristics of the carbonaceous slate in water-rich areas.

## Introduction

With the development of underground structures in the mountainous areas of central and western China, large deformation of the surrounding rock induced by the creep has become a severe problem affecting construction and operation. Excavation causes the rock mass to rupture, making the primary fissures continue to expand or produce new cracks. Meanwhile, it changes the stress state and the groundwater distribution. As an essential environmental factor, water could cause geotechnical engineering disasters, such as tunnel collapse and surface subsidence^1^. Zhao et al.^2^ found that the significant strength reduction of carbonaceous shale under the action of groundwater is the main reason for tunnel asymmetric large deformation. The groundwater stored in the joints and fractures of the rock mass has a significant impact on the mechanical properties, and the creep deformation is particularly remarkable, which will promote the deformation of the rock mass and trigger the instability of the surrounding rock and the supporting structure in a prolonged time^3^. The amount and duration of long-term deformation of the rock mass are related to not only the mineral composition, microstructure, joints, fissures, and structural surfaces but also external factors such as groundwater and stress state, which are especially sensitive to the soft rock represented by carbonaceous slate.

Water-rock interaction is a complex process. The long-term water-rock reaction will reduce the friction and cohesion between mineral particles and change the mineral composition and microstructure of the rock, producing pores, cavities, dissolution fissures, and other defects, so the porosity increases. It causes the deterioration of mechanical properties such as strength and stiffness^4,5^.Olivier et al.^6^ concluded that fluid-rock interactions lead to the evolution of rock’s physical properties and strength. The physical and chemical interactions between fluid flow and tectonic structures, such as fault zones, strongly influence the mechanical behavior of the crust at different space and time scales. Macroscopic fractures and faults are preferred pathways for fluids. From the micro perspective, the mineral component, arrangements of particles, micro-defects, intergranular cracks, and micro-flaws controlled rock mechanical behaviors^7,8^. Jiang et al.^9^ believed that water-rock interaction reopens natural fractures and beddings during spontaneous imbibition, thus enhancing permeability. Yang et al.^10^ characterized the changes in the number, size, and connectivity of micropores and fractures in carbonaceous slate samples during water injection and axial loading using T_2_ spectroscopy of NMR, finding that the poor connectivity between micropores and fissures in carbonaceous slates. Water’s physical and chemical action altered the rock mass’s internal structure and mechanical properties, significantly reducing its load-bearing capacity^11–16^. The mechanisms on which confining and seepage pressures affect a rock’s mechanical behavior have been studied^17–20^. The interaction of pore water with the rock mass manifests in two ways: the physicochemical interaction of water and rock affects the effective stress and deformation of the rock skeleton, and the deformation of the rock mass affects the seepage of water and the pore water pressure^17,21,22^. Seepage pressure affects the elasticity and brittleness of a rock by acting on pores, thus changing the mechanical properties of the rock. The inflow of fluid affects the mechanical behavior of the rock by changing the pore pressure and arrangement of the cementing material^23^. Yang et al.^5^ conducted triaxial compression tests on carbonaceous slate to study the effect of different water saturation times on the microstructure and strength of soft rock. Shen et al.^24^ assessed the evolution mechanism of fracture extension in deep-ground engineering rock under the coupling of fluid-force fields. Song et al.^25^ studied carbonaceous slate by graded incremental cyclic loading and unloading, showing that the effect of water on the carbonaceous slate is mainly manifested in transient plastic strain and viscoelasticity.

Creep is the continuous deformation of rock under constant stress with time. In general, the creep deformation of rocks is characterized by three distinct phases^15,26,27^: in the first stage, the rate of axial strain continuously decreases with time, known as the primary creep phase; in the next stage, the rate of axial strain remains constant with time, referred to as the secondary creep phase, in the last stage, the rate of axial strain exponentially increases with time, called the tertiary (nonlinear accelerated) phase. Some experimental studies have shown that the water considerably impacts the weak rocks’ creep behavior^28,29^. Sun et al.^30^ performed several sets of creep tests on jointed rock bodies and analyzed jointed surfaces’ creep characteristics and damage forms. Zhu and Ye^3^ concluded that water is an essential factor influencing rock creep. Zhang et al.^31^ believed that under the coupling of long-term shear loading and pore water pressure, the mechanical properties of the structural surfaces continuously deteriorate, promoting the creep of the rock mass, which poses a significant safety hazard to the project. Liu et al.^32^ analyzed the creep curves of dry and saturated rock samples by uniaxial creep tests, found that the creep properties differed considerably, and emphasized the indispensable influence of water. Tarifard et al.^1^ demonstrated that the induced stresses are more considerable in the rock surrounding the tunnel due to the rock creep behavior and underground water. Jiang and Wang^33^ discovered that the deformation gradually increases and the long-term strength gradually decreases with the increase of water content by soft rock shear creep, the particles undergo a change from fracture to rotation on the shear surface, and the traditional Burger model was improved based on considering the effect of water content. Wang et al.^34^ carried out experimental research on triaxial compression and creep of carbonaceous slate, analyzed the characteristics of axial and lateral creep under different stress levels according to the creep curve, and proposed the damage creep model. Chen et al.^35–37^ studied the whole process of the water–rock coupling creep and analyzed the deformation law in the creep process of each stage.

Currently, research outcomes mainly focus on the effect of water on the instantaneous mechanical properties of the rock mass. It showed that the softening impact on the rock is related to the species and content of clay minerals, which is more pronounced in soft rocks, leading to a decrease in their mechanical parameters, and that the pore water affects the effective stresses in the rock skeleton. However, few studies have been performed on the effect of water on the long-term mechanical behavior of rocks. In particular, the soft rock represented by carbonaceous slate has a high sensitivity to water in its physical and mechanical properties. Therefore, more research literature on the effect of water on rock creep needs to be conducted from different scales. In this paper, triaxial creep tests were carried out on dry and saturated specimens, and the strain variation rules in the axial and radial directions were analyzed, respectively, considering the effect of layered planes. Then, a comparative analysis of the carbonaceous slate’s microstructural morphology and pore changes before and after creep was conducted using SEM and MIP. The microscopic features such as pores, fissures, and particle state of the specimen can be observed under different magnifications, and the changes in the number, size, and connectivity of the pores of the rock samples before and after creep can also be quantitatively characterized. Hence, the creep deformation mechanism of carbonaceous slate under the action of water was better understood from the microscopic point of view. The qualitative analysis of the carbonaceous slate’s creep mechanism under water’s influence can better reveal the prominent macroscopic deformation characteristics of rock in the surrounding tunnel. The study’s results can provide a complete technical reference for accurate analysis and assessment of the stability of underground engineering surrounding the carbonaceous slate and, at the same time, lay a foundation for the subsequent study of the carbonaceous slate creep constitutive model and its parameters.

## Materials and methods

### Specimens and the Preparation

The specimens, with a natural density of 2.71–2.76 g/cm^3^ and porosity of 1.8-2.36%, were taken from the Muzhailing Tunnel of Lan-Yu Expressway, located in Dingxi City, Gansu Province, China. The surrounding rocks of the tunnel are mainly monoclinic layered carbonaceous slate and sandy slate interbedded. Due to the different content of constituents, the rock mass is dark grey and grey, and part of its laminated surfaces are enriched with sericite minerals and show sericite luster. The joints with remarkable heterogeneity are more developed and softened by rich fissure water. In contrast to sandy slate, carbonaceous slate has lower strength and more significant deformation. Due to some factors such as unloading of tunnel excavation, weathering, and vibration in the sample-making process, it was inevitable that certain damages to the rock specimens produced, which destroyed part of the bonding among the laminar surface of the samples, expand microcracks and other defects exist. It increases the difficulty of making the required specimens. In the specimen processing process, 50 mm diameter cylinders were drilled along the vertical and parallel layers of slates with large rock blocks, and then the samples were processed into standard specimens with a height of 100 mm using a cutting machine and a two-end smoothing machine. To reduce the test error caused by the difference in the internal defects of the rock samples, wave velocity was detected to evaluate the internal pores and damages of the rock samples based on the principle that the propagation speed of acoustic waves in solids is much larger than that in liquids and gases. Wave velocity tests were carried out by applying two acoustic tester probes to the two end faces of the machined cylindrical specimens in their natural state. The results are shown in Table 1. Both compression and shear waves propagate faster in the vertical samples, with average wave speeds of 4392.63 m/s and 3536.20 m/s, while they propagate slower in the horizontal samples, with 2889.71 m/s and 2039.14 m/s, respectively. The wave speeds of the two horizontal samples in the natural state are very close to each other and the same as those of the vertical samples. Carbonaceous slate is a typical transverse isotropic material with different mechanical responses at the perpendicular lamination plane and parallel to the lamination plane. When sound waves propagate along the horizontal rock samples, they have to pass through the intergranular porosity and significantly reduce their wave speeds; when they propagate along the vertical samples, they mainly propagate along the dense solid particles, and the intergranular microfractures have very little influence on the wave speeds, they need to pass through fewer perforated cracks and have higher wave speeds. It indicates that the rock samples’ internal pore structure and the degree of damage are similar. The rock samples with similar wave velocities in the acoustic wave test were selected for the comparison test. Creep tests were carried out on specimens in dry and saturated states to study the mechanism of groundwater influence on the creep properties of damaged carbonaceous slate. Specimens marked H-1 and V-1 saturated in a vacuum-pressurized saturation device (ZYB-II) under the conditions of -100 kPa vacuum and pressurized 5 MPa for 12 h each. During saturation, the rock sample labeled H-1 showed visible cracks in the middle of the specimen due to reduced bonding between the layered planes caused by water absorption and swelling of clay minerals. Specimens H-2 and V-2 were baked in an oven at 105 °C ~ 110 °C for 24 h and used as dry samples. In addition, some blocks from the creeping of the corresponding samples were processed to be used as SEM and MIP specimens.

**Table 1:** The results of wave velocity in different laminar directions (unit: m/s).

Samples	Description	*P*-waves	Average	S-waves	Average	Note
H-1	Laminated surfaces perpendicular to the sample axis	2974.73	2889.71	2120.28	2039.14	Prepared for saturated sample
H-2	Laminated surfaces perpendicular to the sample axis	2804.68		1957.99		Prepared for dry sample
V-1	Lamination surfaces parallel to the sample axis	4374.47	4392.63	3370.49	3536.20	Prepared for saturated sample
V-2	Lamination surfaces parallel to the sample axis	4410.78		3701.91		Prepared for dry sample

### Experimental procedures

**Fig. 1:**
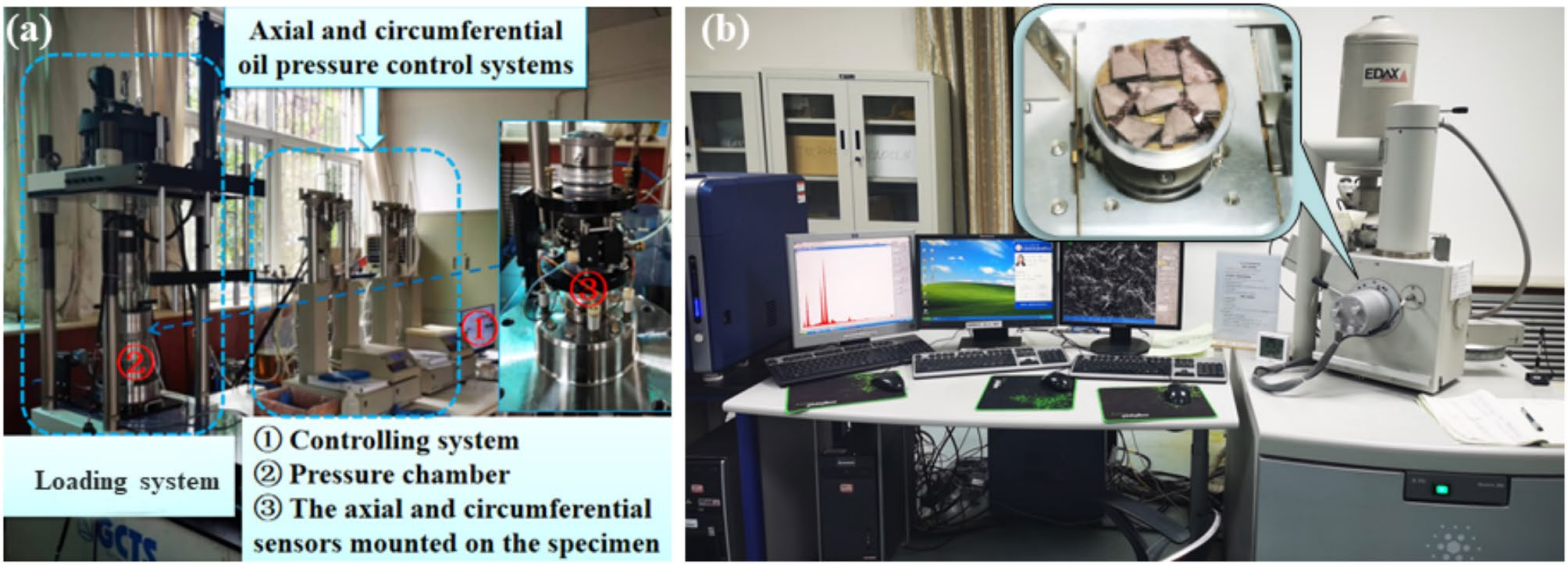
Test equipment and specimen diagrams. (**a**) The GCTS-RTX1000 system; (**b**) Quanta 200 system and the specimens.

Scanning electron microscopy (SEM) is one of the most essential methods to observe the surface morphology of materials from a microscopic point of view. It was used to observe the pores, fissures, and particle characteristics of the laminated surface of the carbonaceous slate specimen at different magnifications. Mercury intrusion porosimetry (MIP) is a method in which mercury is pressed into a solid material at high pressure to detect the pore characteristics of the material. SEM and MIP experiments were used to detect the morphology of the surface and porosity characteristics of the carbonaceous slate. The representative small specimens were selected from appropriate parts of the small blocks retained when the rock samples were cut and the blocks after the creep test. Small pieces of rock samples were broken along the laminar surface, and the fresh side was gold-sprayed and placed under an environmental scanning electron microscope (Quanta 200, FEI) to observe the internal structural morphology of the rock samples. The instrument and the specimen after gold spraying are shown in Fig. 1b. The void characteristics of the specimens were studied using mercury intrusion porosimetry with a MicroActive AutoPore V 9600, American. These two tests are relatively routine experiments, and the specific processes will not be expatiated.

The effect of groundwater on the time-dependent deformation characteristics of carbonaceous slate specimens is essential for the study. The triaxial creep tests on horizontal and vertical specimens were performed using the RTX-1000 Rock Triaxial Rheological Test System from GCTS, USA (see Fig. 1). The system utilizes static and dynamic closed-loop digital electro-hydraulic servo control of strain or stress to perform routine transient and rheological tests at varying confining pressures. The strength values of the triaxial compression test of rock samples with similar wave speeds were pretested as the basis for applying axial loads in a graded manner. The strength of the vertical specimen at the same stress condition was about 0.6 times that of the horizontal specimen at the same stress condition. The creep experiments used a stepwise loading method to study the creep response of the carbonaceous slate under the same stress condition. The confining stress of the horizontal rock samples was set at 3 MPa, and the axial stress increased from 5 MPa with an increment of 5 MPa between two adjacent loads. In addition, due to the low compressive strength of the vertical specimens, the initial creep axial stress was set to 5 MPa, and the axial stress was increased stepwise in increments of 2.5 MPa. The loading speed was controlled to 1kN/minute when applying confining pressure and axial force. The creep time for each stage was set at 24 to 48 h based on the deformation stability of the specimen. The next load was applied at the end of the creep process until creep failure occurred in the sample. All experimental parameters and procedures were written into the control program, which enables automatic loading and data collection processes. The specific test steps are as follows:

1. Installation of specimen. Measure the diameter and height of the rock specimen, then mount the specimen on the base of the slidable pressure chamber, and then use heat-shrinkable tubing to fix part of the pedestal, the specimen, and the top cushion block in a straight line. Finally, use an electric hair dryer to heat dry from bottom to top for a few minutes so that the heat-lodging tube shrinks uniformly and wraps tightly around the pedestal, specimen, and cushion block.
2. Install and debug the sensors. Attach the axial sensor bottom bracket, the loop chain sensor, and the axial sensor top bracket to the specimen’s lower, middle, and upper ends in sequential order. Fix the sensor bracket with two positioning pins and connect the sensor connector accurately to the corresponding data acquisition channel. Adjust the travel of the axial and radial transducers to match the test range. Remove the locating pins and measure the distance between the transducers.
3. Bolt the pressure chamber and the base into one piece, then move it to the loading platform for accurate alignment. Finally, inject the silicone oil and connect the hydraulic line, fill the pressure chamber with oil, and make sure there is no gas in the chamber.
4. Prepare the control program according to the loading scheme and input initial parameters such as rock sample size and axial sensor distance. Ensure that the instrument and the sensor are in good condition, then start executing the program that controls the test processes and collects the relevant data automatically.
5. The accuracy of the experiment is analyzed and monitored based on real-time stress-strain relationships during the test until the protocol run is completed or the rock sample is destroyed. The test is terminated by unloading the sample according to the reverse of the loading procedure and recording the damage pattern of the rock sample after the test.

## Results and discussion

### Characterization of microstructural changes during creep processes

The microstructural variations of carbonaceous slate affect its macroscopic mechanical properties. The SEM obtained the carbonaceous slate’s microstructure features and provided the bedding plane’s accurate geometrical morphology. The pore characteristics within the block after the creep of dry and saturated samples were obtained by MIP testing. The variation in pore size and number distribution and connectivity between the pores can reflect the microstructural changes during the creep process.

**Fig. 2:**
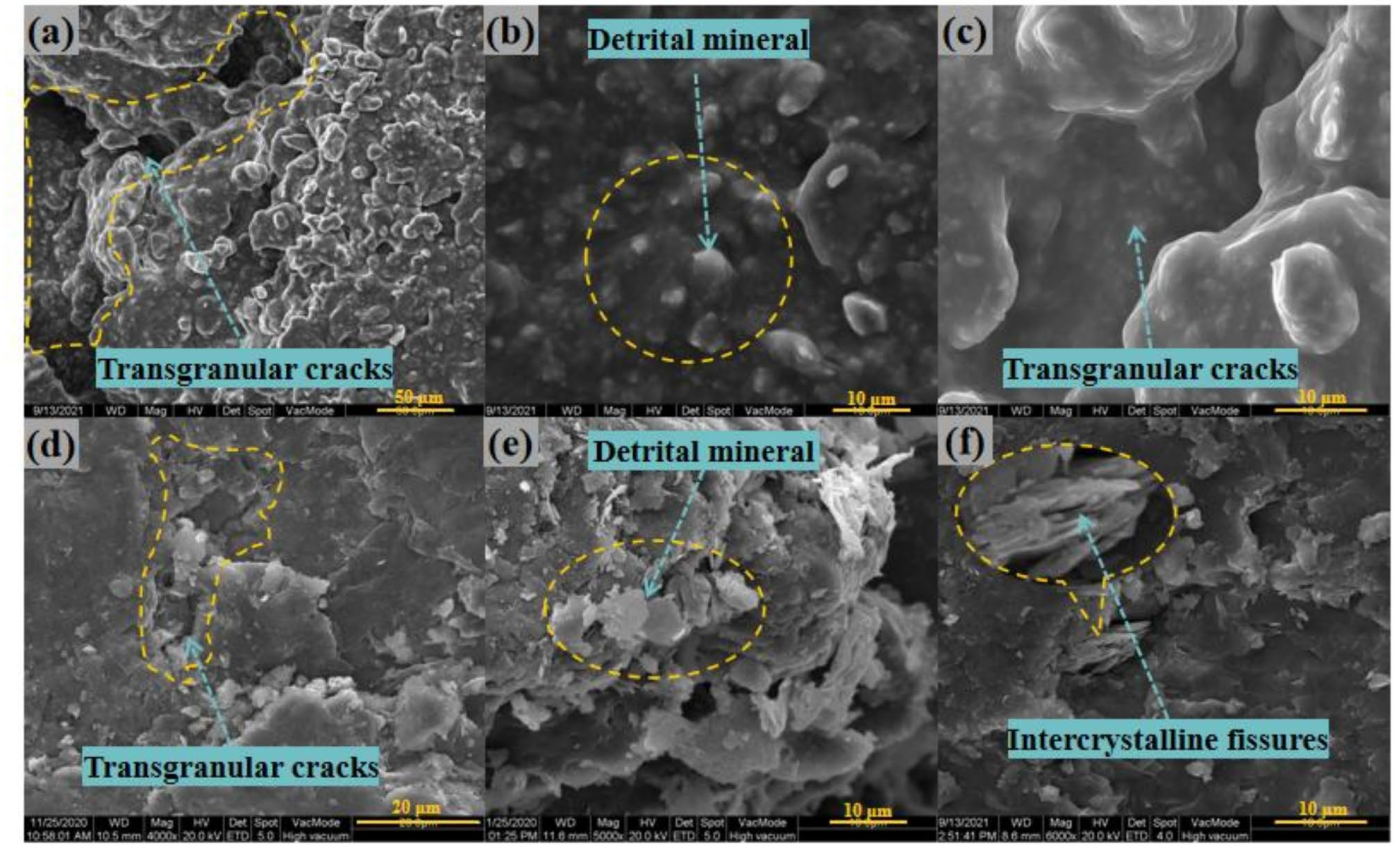
Morphology of the layered surface of rock samples. (**a**–**c**) Saturated samples after creep; (**d**–**f**) Dry samples after creep.

Pictures (see Fig. 2) from SEM were used to evaluate the microstructural differences in particle migration between dry and saturated samples after creep processes. It visually shows that the carbonaceous slate consists of flaky particles orientated along the bedding direction, forming fish scale-like intergranular microporosity, and micro-fissures intersect the bedding plane. The surface morphology of the carbonaceous slate can be observed in the form of laminated granular debris, intercrystalline fissures, and transgranular cracks. Figure 2a and c demonstrate the state of the particles and cracks on the layered surface of a saturated rock sample after creep. The rock samples had been misaligned and partially fractured during the creep process.

The edges of the lamellar particles were rounded by dissolution after the saturated sample underwent creep, the dissolved material filled in between the grains as well as in the per crystalline gaps, and the intergranular microcracks disappeared as a result of the swelling of the clay minerals by water uptake and the deposition of the dissolved material. The fractured surfaces and crack edges among the layered planes of dry rock samples after creep were distributed with disordered groups of detrital minerals, see Fig. 2d and e. In contrast, the shape and distribution pattern of the flaky debris free from the matrix rock in the dry sample was evident, and the angularity of the flaky particles and the transgranular cracks were distinct. The laminar detrital particles were compressed to form a denser, thicker laminated structure, and some intergranular fissures can be observed in the local section. It confirmed that carbonaceous slate has a dense internal structure with poor permeability and water absorption properties. During the creep processes, carbonaceous slate particles misaligned with each other to form macroscopic cracks under the action of shear stress, which changed the porosity and the seepage path. In addition, mineral particles also underwent dissolution, migration, and deposition under the influence of water.

**Fig. 3:**
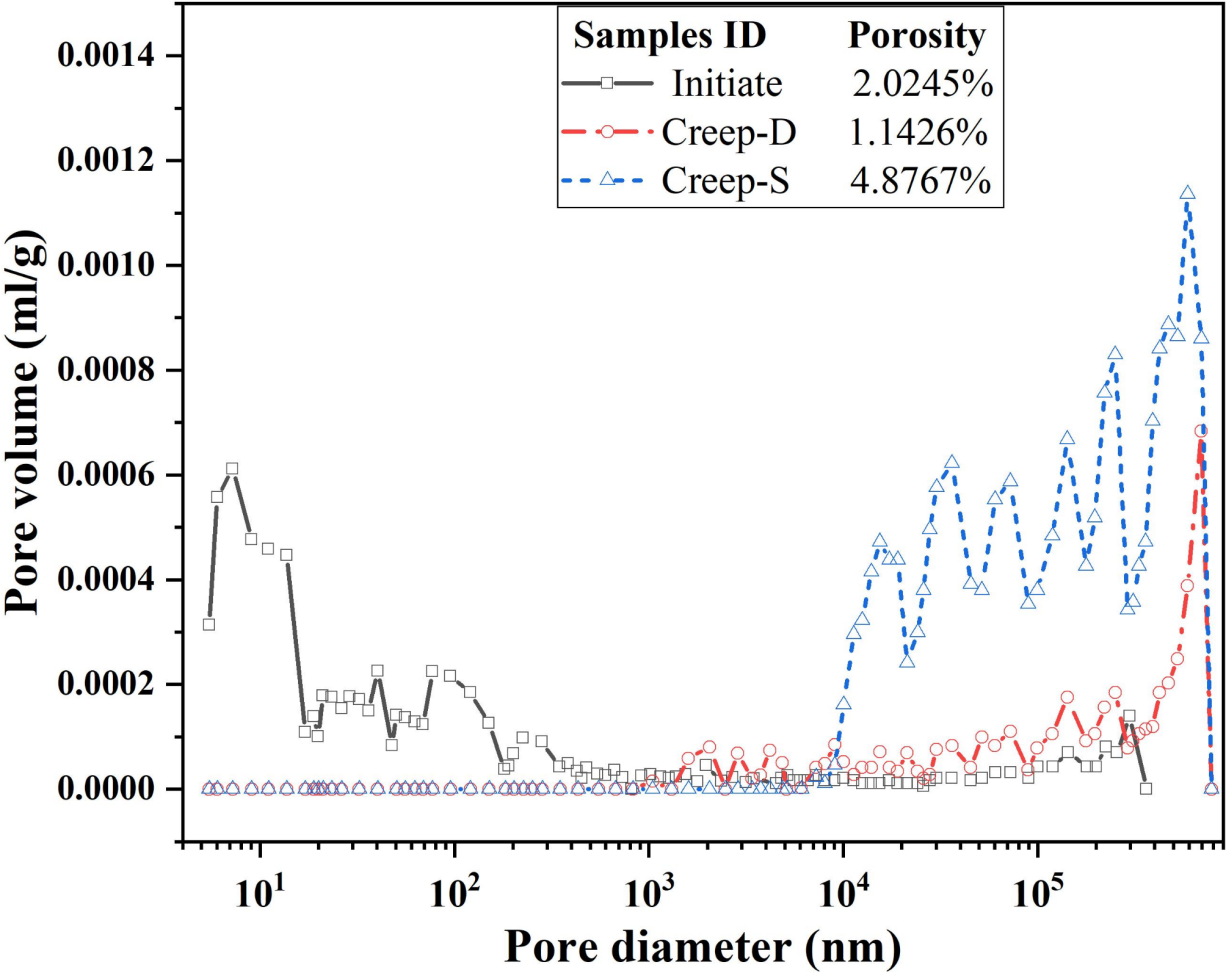
The variation of the specimens’ void volume and the diameter.

MIP tests were carried out on the initial rock samples and the dry and saturated samples after creep to quantitatively analyze the evolution of the internal pore characteristics of the samples during the creep processes. Pore size and distribution of vertical rock samples before and after creep were detected. The results are shown in Fig. 3. The data for the initial natural rock samples is marked ‘Initiate’, and the data for the dry and saturated samples after the creep test are recorded as ‘Creep-D’ and ‘Creep-S’, respectively.

Figure 3 shows the volume amounts corresponding to different diameters of voids per unit mass of the specimen. The pores in the initial natural samples are mainly composed of micropores smaller than 1 μm, with a porosity of 2.024%. After the creep test, the pore diameters of the rock samples were all larger than 1000 nm, and all the original micropores smaller than 1000 nm disappeared. The porosity of the vertical saturated sample was 4.8737%, while that of the horizontal dry sample was 1.1422%. Combined with the SEM images of the specimens before and after creep, it confirmed that the micropores between the specimen particles closed and the pores of the specimens changed into fissures. The pressure dissolution, migration, and deposition occurred at the edges of the saturated sample particles, filling some of the voids at the microfracture tips. In addition, the change in porosity reflects the different deformation of specimens with varying angles of bedding. Horizontal rock samples exhibited compaction, while vertical rock samples formed tension and shear cracks due to grain misalignment.

### Creep deformation characteristics of dry and saturated samples

Carbonaceous slates are metamorphic rocks with flaky formations. The presence of bedding planes leads to different mechanical responses to water and stress. According to the various angles between the layered plane and the axis of a cylindrical specimen, all the rock specimens were roughly divided into horizontal and vertical samples. A constant confining compressive stress (σ_3_) is applied to the sides of the cylindrical specimen, and stepwise increasing axial compressive stresses (σ_1_) are applied to the ends of the cylindrical specimen during the creep processes. The deviator stress (σ_D_) value showing above the strain curve at each stage is expressed as follows: σ_D_ = σ_1_- σ_3_. The axial and radial strain curves with time during the test are as follows:

**Fig. 4:**
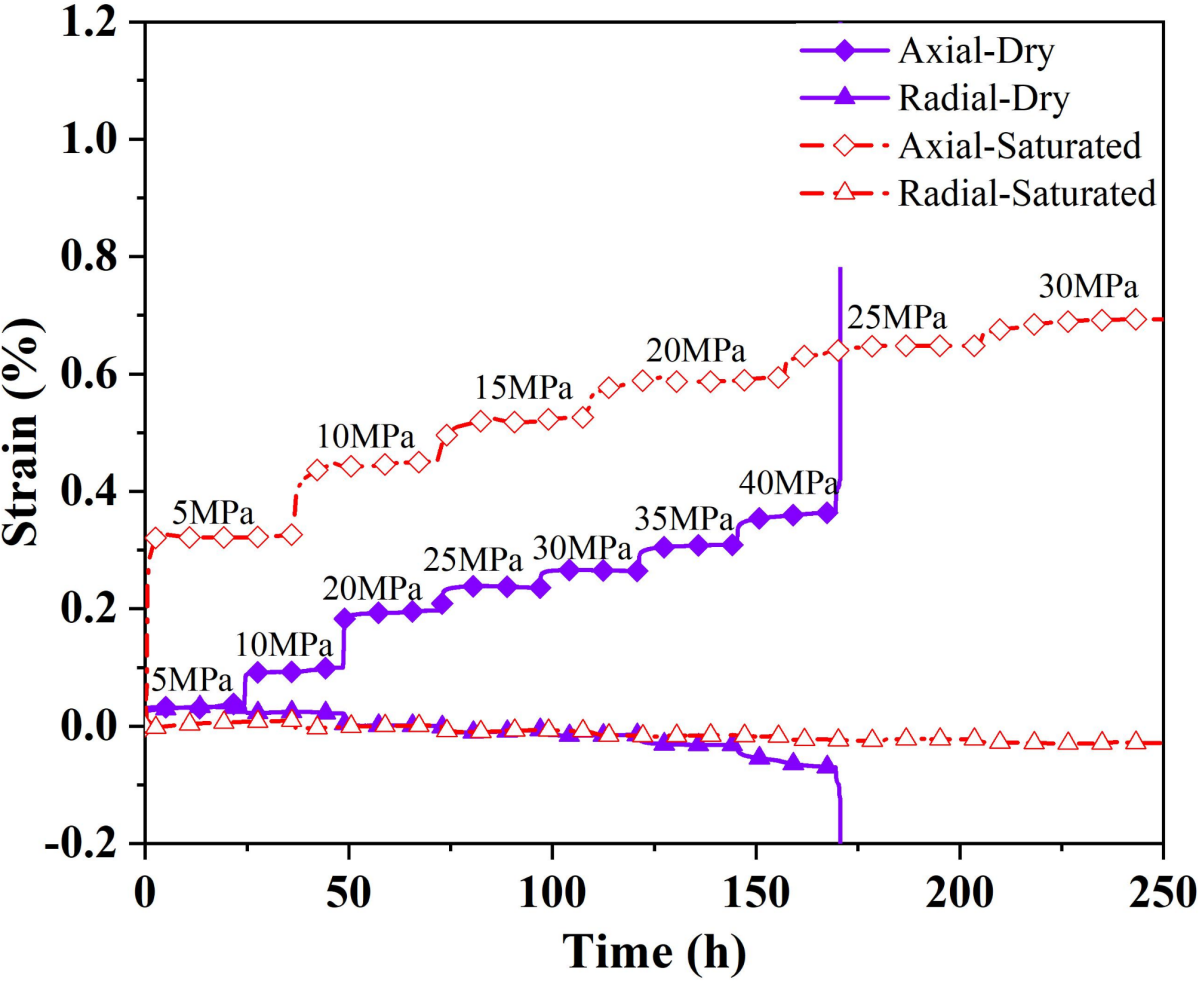
Strains versus time during creep test of the horizontal sample.

**Fig. 5:**
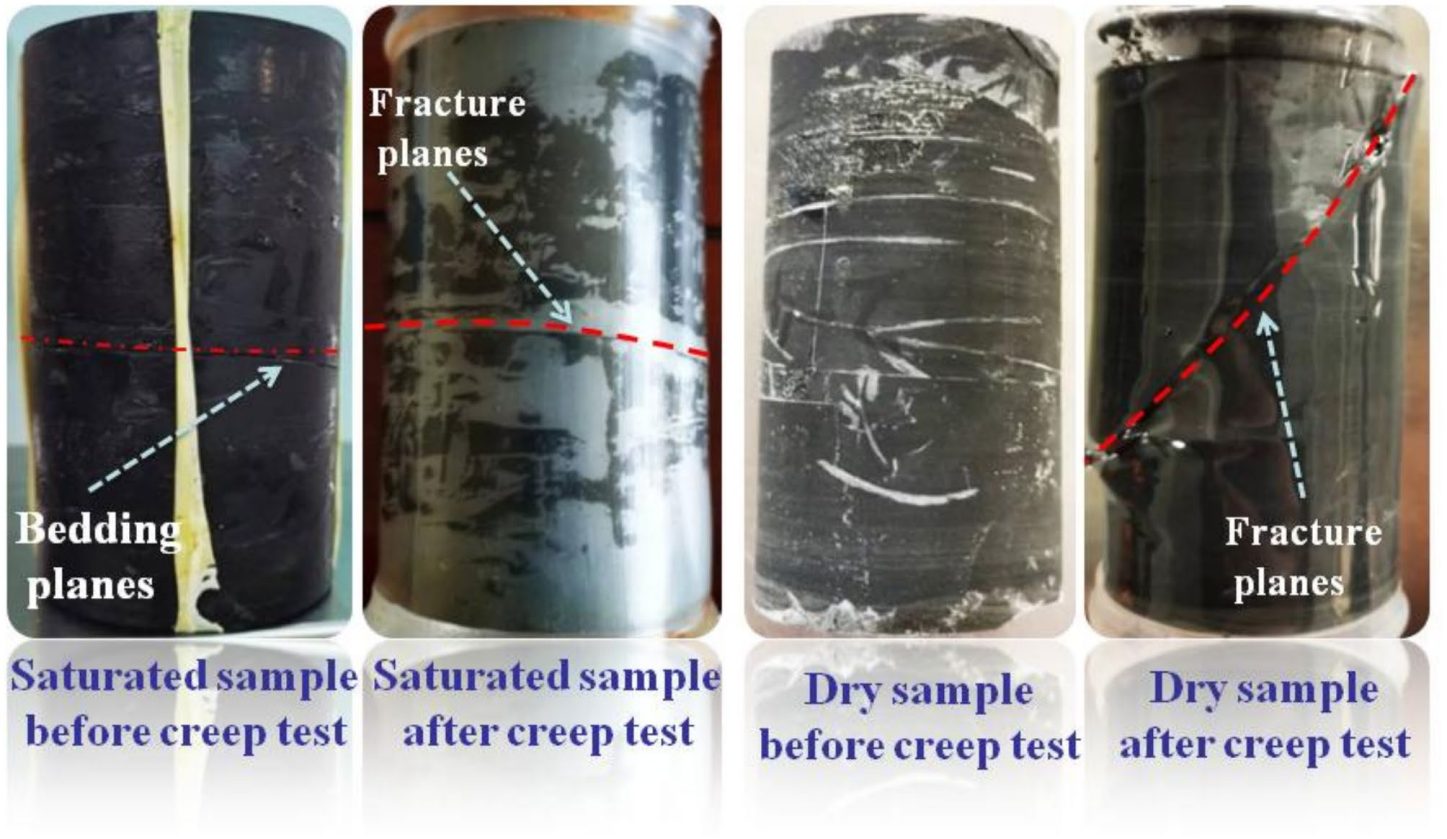
Failure pattern diagram of horizontal samples.

**Fig. 6:**
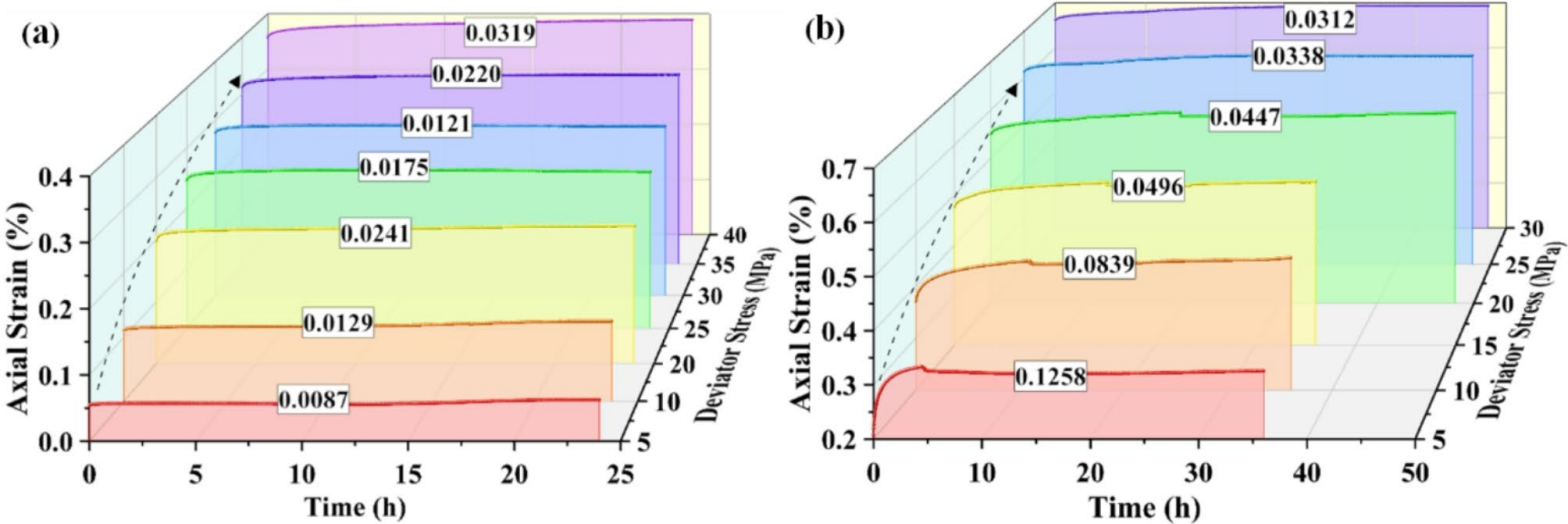
Axial creep with time for horizontal specimens under different deviator stress. (**a**) Dry sample; (**b**) Saturated sample.

Figure 4 displays the variation of strain overtime during the creep experiment. The rock samples all show typical creep characteristics during the creep process. With the increase of the deviator stress, the change of each creep curve is geometrically similar, dominated by transient creep and steady creep. The saturated horizontal specimen in the test process is still not destroyed and has not yet shown an accelerated stage after the deviator stress reaches a set maximum value of 30 MPa. Dry horizontal specimens in the early creep stage also showed the first two stages. When the deviator stress reached the peak stress (42.5 MPa) destruction, the curve still had no noticeable creep accelerated stage. While in a very short period, the strain increases dramatically, showing significant brittle damage. The instantaneous deformation produced by the saturated horizontal specimens at the initial application of external load was more significant than that of the dry specimens, with an axial strain increment of 0.0466% and a radical strain increment of 0.0312%, which were 2.34 and 1.18 times higher than those of the dry specimens, respectively. The vacuum pressurization saturation process makes water fully enter the porous space of rock samples. The peak stress and the form of damage of rock samples in triaxial compression tests depend mainly on the magnitude of the circumferential and deviatoric stresses, which are also affected by the pore water content of the samples. Figure 5 presents the changes before and after the creep of the horizontal samples in both dry and saturated states. During the water saturation process, the saturated sample had visible cracks due to the water absorption and expansion of clay minerals on the layered planes. It did not enter the damage stage even after the deviatoric stress reached 30 MPa during the creep process. However, the dry sample destroyed and deformed a typical shear rupture interface with a large angle to the layered plane at smaller confining pressures and larger deviatoric stress states. The pore water pressure formed by the absorption of water in the microporous space shared the stress of the particle skeleton. It improved the ability of rock samples to resist external loads. The physical effect of water rock makes the rock sample particles soften, reducing the deformation modulus as well^38^. The strain changes at each stage of the creep process are demonstrated in Fig. 6.

The creep process of both dry and saturated samples experienced initial creep and stable creep stages, while the deformation amount and characteristics were different at different stress levels. In the early stage, the dry sample was in a low-stress state, the strain curve mainly showed stable creep, and the initial creep stage was inapparent. With the increase in the stress level, the initial creep was gradually highlighted. The creep tended to increase (see the value shown by the dotted line in the Fig. 6). It can be seen that the creep increments of the dry rock samples in the elastic phase were about 0.01% and 0.0241% in the pore closure stage. The development of cracks before the destruction of the initial creep stage was notable. However, the creep amount of saturated samples decreased from 0.1258 to 0.0312% with the increase in stress level. The saturated sample creep curve had a significant tendency to nonlinearly increase in the initial creep stage and decrease with the rise in stress level. The sudden change in strain during the process can be inferred as damage to the specimen, which occurred along the internal cracks. The creep amount of the saturated rock samples is more considerable than that of the dry samples, and the creep of the saturated samples is 14.5, 6.5, 1.85,1.9 times that of the dry samples in the same stress state(5 MPa, 10 MPa, 20 MPa, and 25 MPa, respectively), which decreases with the increase of the stress level.

**Fig. 7:**
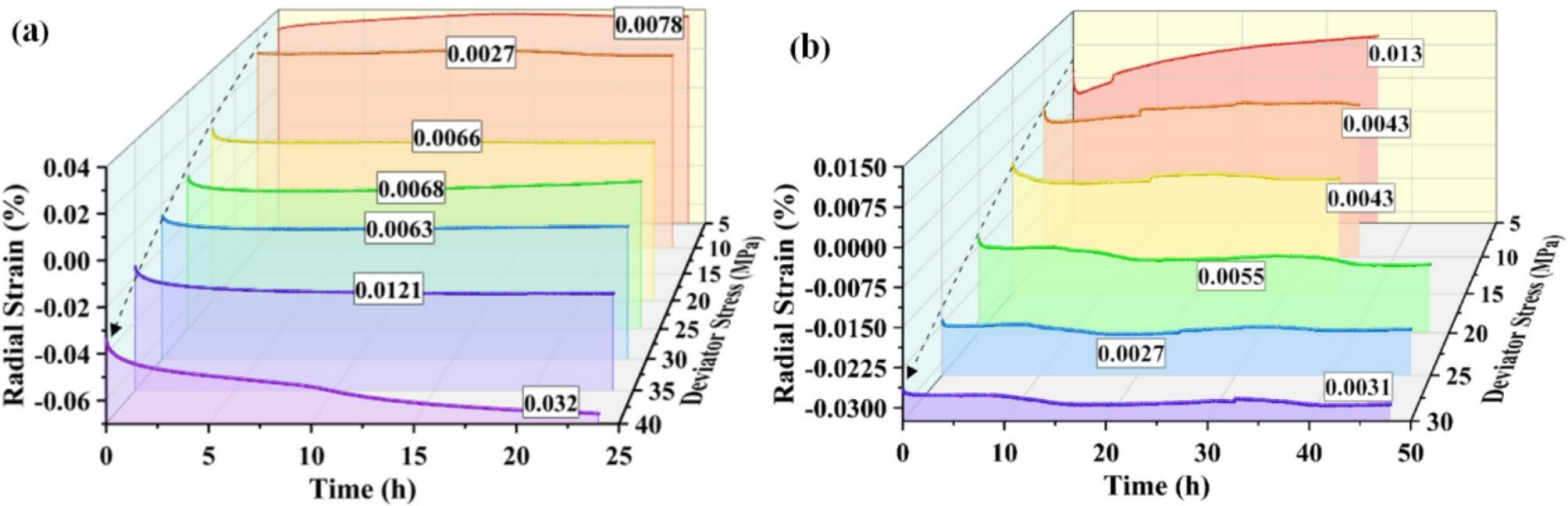
Radial strain with time for horizontal specimens under different deviator stress. (**a**) Dry sample; (**b**) Saturated sample.

The radial creep curves of the horizontal samples are shown in Fig. 7. The creep curves of the dry samples are smooth, and the strain changes are continuous and stable, while the curves of the saturated samples fluctuate, and sudden changes occur locally. It can be well explained by the fact that horizontal rock samples broke the bond and formed macroscopic cracks during the saturation due to water absorption and expansion of minerals on the layered planes. The dislocation of particles within the cracks occurs during creep. With the increase of deviator stress, the radial creep of the specimen gradually declined from the initial positive value to the negative value at the later stage. The radial creep of the dry sample tended to decrease from 0.0078 to 0.0027% and then rise to 0.032% as the stress level increased. While the creep of the saturated sample declined from 0.013 to 0.0031%, the lack of increase stages is because the rock samples have not yet entered the phase of fracture development. At low stress, the radial creep of the saturated sample is greater than that of the dry sample (about two times) and less than that of the dry sample at high stress. Radial creep was less than axial creep. It inferred that the rock samples were filled with water in the pores between the layers during saturation, and the physical interaction between the water softened the rock samples and reduced their deformation modulus. The presence of pore water made the deformation last longer and significantly affected creep in the low-stress stage, where porosity was large.

**Fig. 8:**
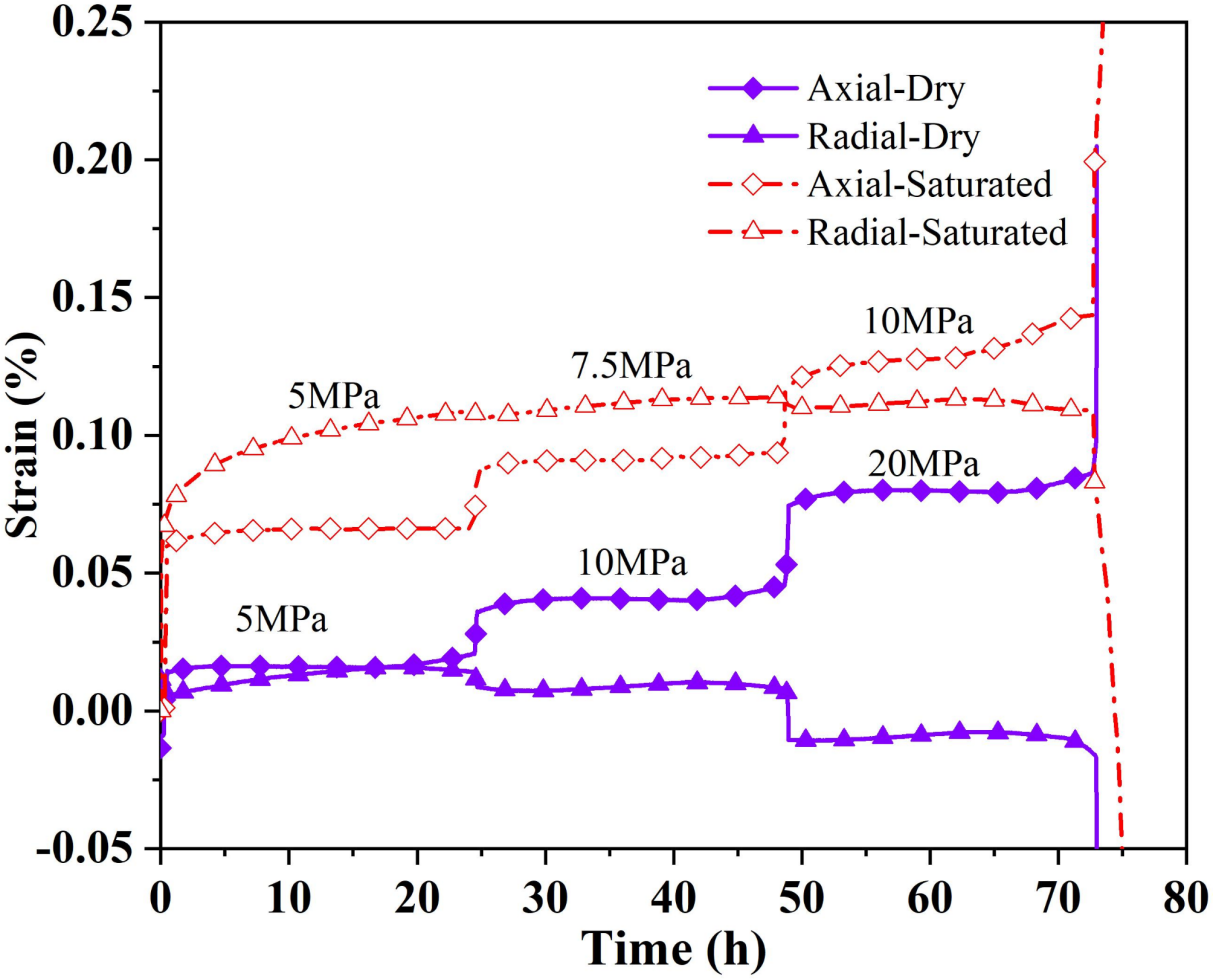
Strains with time of vertical samples.

Since the strength of the vertical rock samples was less than that of the horizontal samples, and the strength of the saturated samples was less than that of the dry samples, the deviator stress increments of the specimens were appropriately adjusted during the tests. In the experiments, four loadings and three creep courses were experienced. Finally, the saturated samples were damaged after creeping for about three hours at a bias stress of 12.5 MPa, while the dry samples were broken when applying a bias stress of 25 MPa. The data of the creep processes are shown in Fig. 8.

**Fig. 9:**
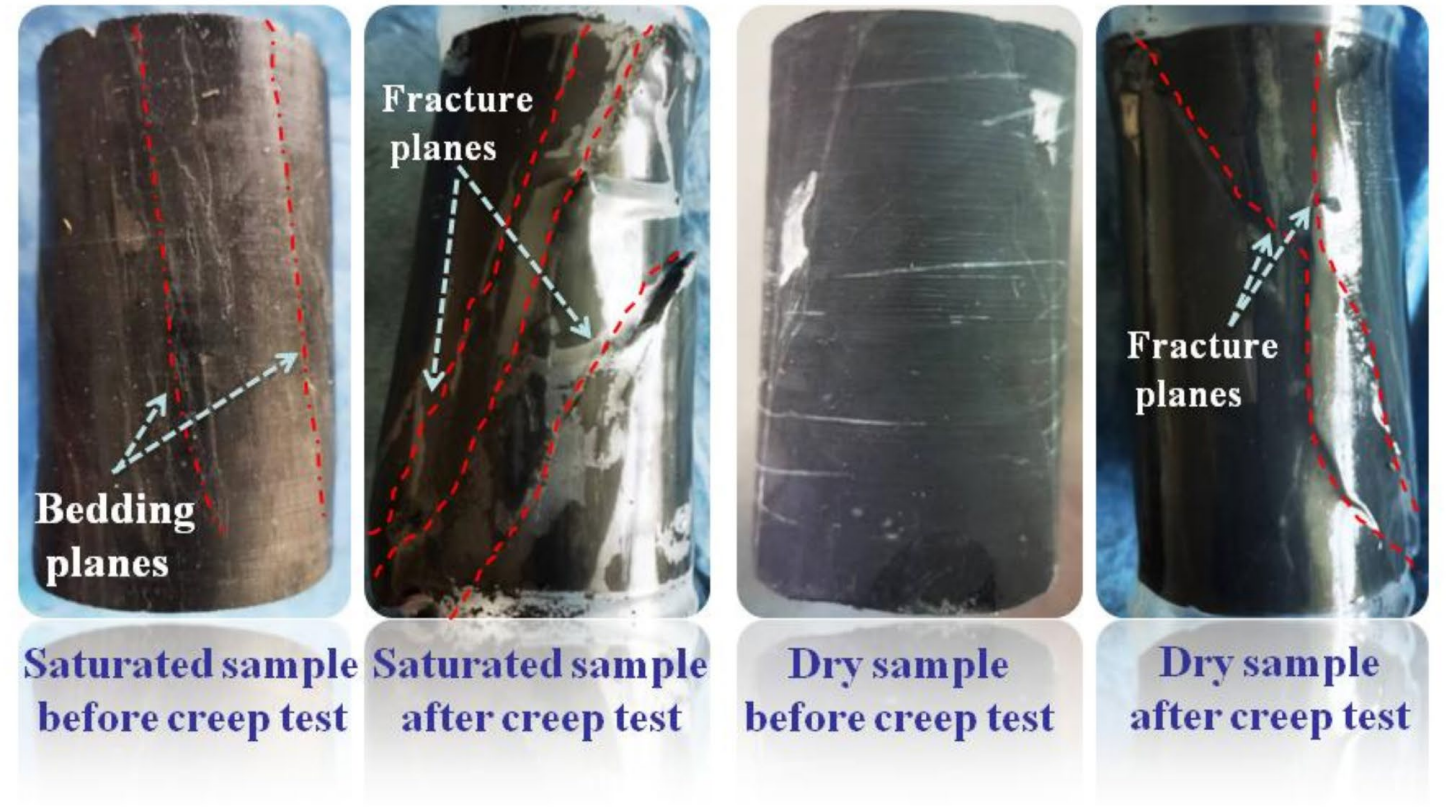
Failure pattern diagrams of vertical rock samples.

**Fig. 10:**
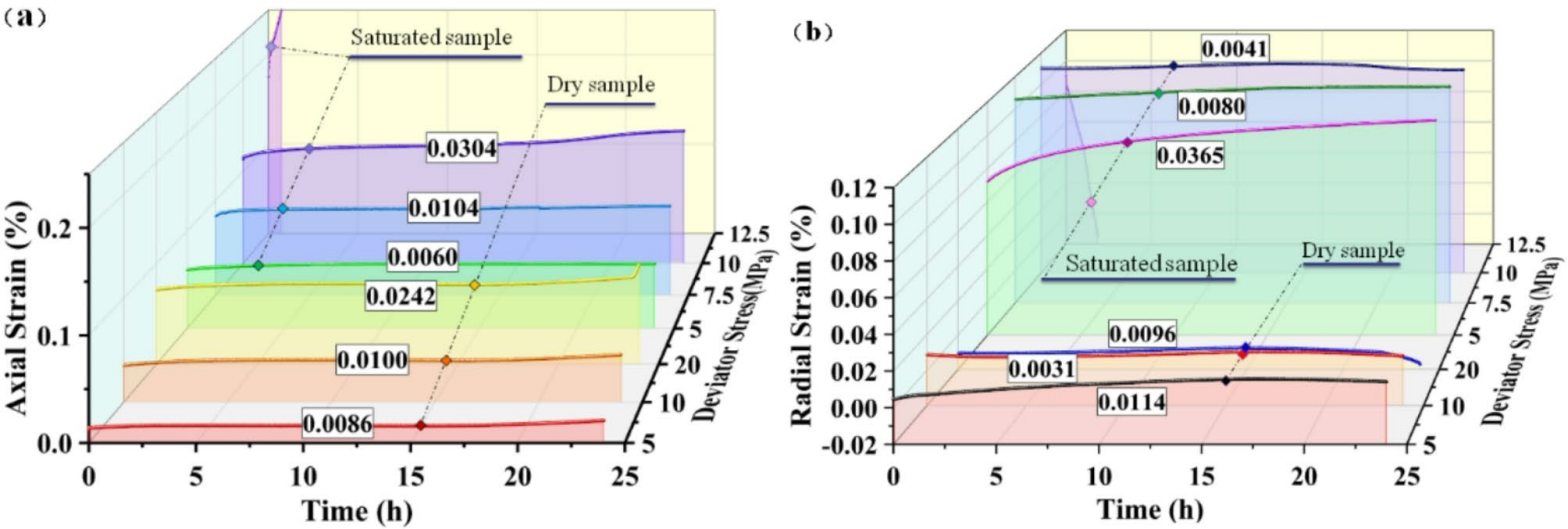
Creep with time for vertical specimens under different deviator stress. (**a**) Axial strains; (**b**) Radial strains.

During the initial application of 3 MPa of surrounding pressure and 5 MPa of deviator pressure, the instantaneous strain increments of the saturated samples are more prominent than those of the dry samples both in the axial and radial strain(about five times). Both rock samples exhibit initial creep during the first creep at lower stress levels, with the radial creep being more pronounced and lasting longer in the saturated samples. The creep amounts were smaller in both the second creep. While the saturated samples showed a significantly accelerated phase in the third creep, they could still withstand continued loading until they entered the fourth phase and then quickly destroyed and failed. The maximum axial strain at destruction was 0.145% and 0.087% for saturated and dry samples, respectively, and the radial strains were 0.1091% and − 0.0151%. Figure 9 exhibits the patterns of vertical rock samples as they undergo creep failure in the dry and saturated states. The shear failure of the rock samples occurs under the combined action of axial force and radial stress during the creep process. During the saturation, water-rock action destroyed the bonding properties of the bedding planes of the rock sample, and several near-parallel rupture surfaces appeared. The bonding properties between the bedding were weakened so that rupture developed essentially along multiple parallel weakly layered planes, and the rupture surfaces were relatively smooth. The rupture interface of the dry samples was not flat and consisted of a combination of fracture surfaces and bedding planes. The transient strains subtracted during loading from the above curves and the time-dependent strains of the specific creep processes are shown in Fig. 10.

From the strain versus time curves of the vertical specimens in the creep experiments, the water not only reduced the strength of the rock samples but also had a significant effect on both the transient deformation and creep deformation of the rock samples. The axial creep of dry and saturated samples tends to increase with axial stress. At low stress levels, it was dominated by a stable creep with strain increments of 0.006% and 0.0086%. At high-stress levels, the saturated samples (10 MPa) showed a significant strain increase(0.0304%) in the late period of axial creep. As the axial stress rises, the radial creep of the saturated sample decreases, while the radial creep of the dry sample declines from 0.0114 to 0.0031% and then increases to 0.0096%. When a bias stress of 12.5 MPa was applied, the saturated rock samples entered the creep acceleration stage, and the damage occurred after a sharp increase in axial strain for about three hours, which showed the characteristics of ductile damage. The dry samples underwent a sharp augment in apparent axial strain during the last two hours of the creep period at 20 MPa. Finally, the dry samples broke during the application of axial stress, exhibiting brittle damage characteristics. The amount of axial creep increased with the increase of stress. The compression of solid particles mainly caused the axial deformation of the vertical sample. The tendency of the radial creep amount to decrease with the increase of axial pressure and the cyclic strain of vertical rock samples was greatly influenced by pore deformation and pore water. In the low-stress state, the initial creep stage of the saturated samples in the ring direction is very significant (5 MPa bias stress). Due to creep acceleration in the late stage of creep at 20 MPa, the creep amounts of the dry sample are more significant than that at 10 MPa.

### Mechanistic analysis of the effect of water on the creep of carbonaceous slates

Carbonaceous slate is a slightly metamorphosed sedimentary rock with a layered structure. It comprises lamellar particles and fish-scale cleavages and fractures, forming a dense layered structure. During saturation in a low or no-stress state, water fully enters into the internal voids of the rock samples, and the initial rock samples undergo expansion and deformation due to water absorption in the pores. In the creep test, the softening effect of water reduces the strength and deformation modulus of the rock samples, and it significantly impacts the transient deformation of the rock samples^4,38^. The dissipation of pore water under the action of external forces requires a certain amount of time, and the proportion of particle skeleton stress increases gradually with time in the creep process. Therefore, The strain changes in the first creep process were the largest, and the creep deformation showed a gradually decreasing trend, which is related to the connectivity of the pores of the rock samples. The comparative analysis of axial and radial strain under different laminar angles showed that the creep deformation of rock samples in various directions was related to the void deformation characteristics. The particles bent under the force perpendicular to the bedding plane, and the voids compressed significantly. The columnar structure between adjacent bedding was subjected to axial pressure under the action of external force parallel to the bedding surface, and the particles underwent compressive deformation, which has less effect on the voids. During the experiments, the rock samples experienced the formation of maximum densities after the closure of micropores. Then cracks appeared and developed with increased stress until the rock samples were damaged. Compared with the dry samples, the saturated samples deformed significantly during the pore closure stage and showed more obvious creep acceleration and ductile damage characteristics before destruction.

The results of this research can reasonably and scientifically explain the phenomenon of significant large deformation in the water-rich region during tunnel excavation. The excavation of the tunnel disturbances changed the high-stress state and unbalanced the surrounding rock, thus causing rock damage and secondary fissures. Groundwater fills the rock void through the damaged fissure, and the minerals absorb water and expand while softening the rock, making the strength and deformation parameters of the rock samples lower. As the stresses in the surrounding rock continued to adjust during construction, the pore volume was altered to accommodate the external stresses by draining and filling the void water volume. In this process, the mineral debris on the laminar surface was continuously dissolved, deposited, and migrated in the micro fissures under stress and water, forming larger voids and promoting the expansion of rock deformation. However, due to the natural samples formed in a complex geological environment, the mineral composition and the amount of distribution were not uniform, and the dispersion of the strength and deformation values of the rock samples under the action of water was large. The loading increment and number of times are not easy to control, and the creep failure characteristics of the different bedding angles samples could not be observed.

## Conclusions

Groundwater usually induces large deformation of the surrounding rock body and underground structure and even causes disasters such as structural collapse. Carbonaceous slate is a widely distributed soft rock, and its engineering properties are sensitive to water. It brings serious challenges to the construction of underground structures. Hence, researching the creep deformation mechanism of carbonaceous slate under the action of water was put forward. In this paper, SEM, MIP, and triaxial compression creep tests were performed on the saturated and dry specimens of carbonaceous slate. The characteristics of strain curves in the creep process of the specimens before and after creep were analyzed from the perspectives of morphological features such as the size and distribution of particles, micropores, and micro-fissures on the laminated surfaces, as well as the size, number, and connectivity of pores. The deformation process and mechanism of the carbonaceous slate under the combined action of water and pressure were further revealed. The following conclusions are summarized:

1. The effect of water on the mechanical behavior of carbonaceous slate was presented in two aspects: the short-term effect was characterized by its softening effect, which reduced both strength and stiffness and increased the deformation; the delayed deformation also demonstrated the long-term effect, and the size of the deformation was related to the number, the shape and the connectivity of the pores.
2. Intergranular micropores of less than 1 μm dominate the microscopic pores of carbonaceous slate and contain several micro-fissures. The capillary water absorption feature is significant, and the saturated specimens’ initial transient deformation and creep deformation was more prominent due to water absorption and expansion in the unstressed or low-stressed state.
3. In the process of triaxial creep, the rock samples underwent the process of pore closure, densification, and new crack development. The influence of water on the deformation of rock samples was mainly in the pore closure stage. Since carbonaceous slate is a dense laminated structure, the connectivity of the pores is poor, so under the continuous action of external stress, the pore water discharge by extrusion is a slow process at the beginning of the creep, and the pore water shares part of the stress. Therefore, the characteristics in the initial stage of saturated rock samples were significant. As the stress level enlarged, the creep of samples tended to rise, and creep deformation increased dramatically when the specimen entered the destruction stage.
4. Comparative analysis of the changes in the microscopic morphology and pore size and number of the laminar surface of the rock samples before and after creep revealed that the rock samples in the creep process underwent not only particle dislocation and tearing but also pressure dissolution-migration under the action of water as well as deposition under the action of stress. The micropores in the creep process all closed and disappeared, transforming into larger cracks and fissures.

## Data Availability

The datasets used during the current study are available from the corresponding author upon reasonable request.
